# Supplementary material for: Homeostasis after injury: How intertwined inference and control underpin post-injury pain and behaviour
Source: PLoS Comput Biol. 2026 Jan 22;22(1):e1013538. doi: 10.1371/journal.pcbi.1013538 (PMC12851502; doi:10.1371/journal.pcbi.1013538)
Supplement: Attachment — Submitted filename: Cover Letter and Response to Reviewers.pdf [file pcbi.1013538.s002.pdf]

Response to Reviewers: PCOMPBIOL-D-25-01015

Dear Editors and Reviewers,

We are grateful for all of the suggestions, and we thank you for your input. Please find below our point-wise responses in blue, and we have accordingly edited the manuscript as well. In addition, we have also added an author summary as requested.

Sincerely,
Pranav Mahajan
Corresponding author

Reviewers' comments:

Reviewer's Responses to Questions

Comments to the Authors:
Please note here if the review is uploaded as an attachment.

Reviewer #1: This paper extends Seymour et al. (2023) by implementing a formal POMDP-based model in which the brain infers injury states to guide behaviour. The authors use simulations to show that rational behaviours emerge when observations are informative, while maladaptive outcomes (such as chronic pain) arise from information restriction or aberrant priors. This generative modelling framework offers a computational approach to understanding injury-related pain and recovery.

The paper presents two central findings. First, it shows that even when investigating an injury is painful, such behaviour can be rational and beneficial. Within the proposed computational model, this kind of action helps reduce uncertainty about the injury state, allowing the brain to make better long-term decisions about whether to rest or resume activity. Second, the authors demonstrate how chronic pain can emerge through two distinct pathways: either by avoiding information-gathering actions due to fear of pain, leading to persistent uncertainty, or by starting with incorrect prior beliefs about the severity of the injury. Both scenarios can result in prolonged maladaptive behaviour, offering a formal explanation for the transition from acute to chronic pain in the absence of ongoing tissue damage. These findings are intuitive, and they resonate with common sense understandings of how we behave when hurt.

I have previously reviewed this paper as a CCN conference paper, and my earlier comments have been addressed in the present version. My only additional comment is

a suggestion to add a more informative README file in the GitHub repository, and to ensure that all code is clearly commented, as this is not the case at the moment.

Author response: Many thanks for your comments and suggestions. We have now improved the GitHub repository -

- Made the README file more informative explaining which files generate which outputs and mentioning the dependencies
- Added comments throughout the code. Every main function and script is now provided with at least one comment describing the arguments and main variables and what it does.
- Additionally, provided a basic Google Colab notebook which allows readers to generate a basic result in their browser and experiment with it without having to download any code.

Reviewer #2: The article discusses a formalisation of the state of injury in terms of a POMDP; which allows to gain insights into the behavioural processes to aid with recovery, and into how these are shaped by uncertainty about the underlying latent injury state. The paper is well written and well structured. It also acknowledges it being a first step in a somewhat complex and multifaceted modelling endeavour. I think this is an excellent contribution and would appreciate this being published on this outlet. I only have two conceptual notes.

(1) One remark concerns the apparent elementary nature of the model, which penalises its potential for prediction outside of the running example. Specifically, acting usually falls along a spectrum of intensity: take for example the issue of someone who professionally trains e.g. grip strength and needs to both recover from an injury, but also keep their strength from degrading (because it is functional to their career). Their choice becomes one about training intensity (e.g. when to train, and how much training to do). Could the authors perhaps discuss how the model would extend further beyond punctate actions and deal with a dimensional action range? and perhaps trace a connection to the computational theory of vigour ?

Author response: Thank you for this very thoughtful suggestion. We agree this is a limitation of the rather spartan/elementary nature of the model and should be addressed in subsequent future work. Below, we add some discussion points (Line 244-247) on how this could be extended *(edits denoted in italics).*

The model is just a first step. In particular, we did not include transitions in internal states, and thus actual recovery or exacerbation of injuries. *Incorporating this could allow us to specify multiple 'activity' actions on a spectrum of intensity, with varying*

probabilities for worsening one's internal state, differential costs for acting more quickly, intensely or vigorously, and different times of completion. This will allow us to relate our model to notions of activity pacing, i.e. choosing appropriate intensity and timing, and also theories of vigour (Niv et al., 2005, 2007).

(2) Another conceptual note is that while the approach presented is definitely sufficient to account for known phenomena, which of its components are necessary and critical remains slightly unclear. For instance it would be lovely to see the present framework contrasted with a fully model-free approach to learning about the state of injury. One possibility would just be to use a bare actor critic framework, much like Maia's actor critic to explain avoidance conditioning (Maia, T. V. (2010). Two-factor theory, the actor-critic model, and conditioned avoidance. Learning & behavior, 38(1), 50-67.) just revamped for this specific context. What would be the phenomena that are not explained (or explained poorly) in a fully model-free context that are instead well captured here?

Author response: Thank you for another very thoughtful comment. In this work, we are committed to the outcome of the calculations (i.e., the ultimate behaviour) rather than the method of calculation. Therefore, we have provided a computational analysis of the issues rather than an algorithmic one, and would be perfectly happy for a suitable model-free realisation. Your points are very well taken, but appear a little orthogonal to the main contribution. We have attempted to provide further explanation by adding the following paragraph in the discussion (Line 258-268, *edits denoted in italics*).

The core contribution of this work is at the computational level, rather than an algorithmic one (Marr, 2010). We use a model-based approach in this work, which is sufficient to account for the proposed phenomena. Model-free approaches, such as the actor-critic framework (Maia, 2010; Moutoussis et al., 2008), may be able to reproduce the results, provided one uses recurrent neural networks (RNNs) as function approximators. These can learn to represent belief information correctly as long as the RNN capacity is sufficient (Hennig et al., 2023). However, a large amount of interaction data under different scenarios is typically required to train these RNNs and allow them to operationalise the value of information gain, e.g. meta-learning (Wang et al., 2016, 2018). This data requirement presents a challenge, as taking precarious actions (e.g., repeatedly acting when severely injured) risks worsening the injury or, in the worst case, causing death, which would preclude learning altogether. Therefore, it is likely that we are endowed with innate models that are either fine-tuned through experience or used to guide model-free learning. Note further that in the case of actor-critic models, only the critic units would be expected to represent the value of information gain.

We thank the reviewers again for their time and thoughtful comments and hope our responses address them.
